# Age-related changes in olivocochlear efferent innervation in gerbils

**DOI:** 10.3389/fnsyn.2024.1422330

**Published:** 2024-06-03

**Authors:** Friederike Steenken, Asli Pektaş, Christine Köppl

**Affiliations:** ^1^Department of Neuroscience, School of Medicine and Health Science, Carl von Ossietzky Universität Oldenburg, Oldenburg, Germany; ^2^Cluster of Excellence “Hearing4all”, Carl von Ossietzky Universität Oldenburg, Oldenburg, Germany; ^3^Research Centre Neurosensory Science, Carl von Ossietzky Universität Oldenburg, Oldenburg, Germany

**Keywords:** hearing, auditory, cochlea, inner ear, aging, sensory, hair cells, immunohistochemistry

## Abstract

**Introduction:**

Age-related hearing difficulties have a complex etiology that includes degenerative processes in the sensory cochlea. The cochlea comprises the start of the afferent, ascending auditory pathway, but also receives efferent feedback innervation by two separate populations of brainstem neurons: the medial olivocochlear and lateral olivocochlear pathways, innervating the outer hair cells and auditory-nerve fibers synapsing on inner hair cells, respectively. Efferents are believed to improve hearing under difficult conditions, such as high background noise. Here, we compare olivocochlear efferent innervation density along the tonotopic axis in young-adult and aged gerbils (at ~50% of their maximum lifespan potential), a classic animal model for age-related hearing loss.

**Methods:**

Efferent synaptic terminals and sensory hair cells were labeled immunohistochemically with anti-synaptotagmin and anti-myosin VIIa, respectively. Numbers of hair cells, numbers of efferent terminals, and the efferent innervation area were quantified at seven tonotopic locations along the organ of Corti.

**Results:**

The tonotopic distribution of olivocochlear innervation in the gerbil was similar to that previously shown for other species, with a slight apical cochlear bias in presumed lateral olivocochlear innervation (inner-hair-cell region), and a broad mid-cochlear peak for presumed medial olivocochlear innervation (outer-hair-cell region). We found significant, age-related declines in overall efferent innervation to both the inner-hair-cell and the outer-hair-cell region. However, when accounting for the age-related losses in efferent target structures, the innervation density of surviving elements proved unchanged in the inner-hair-cell region. For outer hair cells, a pronounced increase of orphaned outer hair cells, i.e., lacking efferent innervation, was observed. Surviving outer hair cells that were still efferently innervated retained a nearly normal innervation.

**Discussion:**

A comparison across species suggests a basic aging scenario where outer hair cells, type-I afferents, and the efferents associated with them, steadily die away with advancing age, but leave the surviving cochlear circuitry largely intact until an advanced age, beyond 50% of a species’ maximum lifespan potential. In the outer-hair-cell region, MOC degeneration may precede outer-hair-cell death, leaving a putatively transient population of orphaned outer hair cells that are no longer under efferent control.

## 1 Introduction

Age-related hearing loss is one of the most prevalent sensory disorders, affecting nearly one third of the world’s population aged 65 years or older (Liberman, 2017; WHO, 2021). In our society, which is demographically growing older, an increase of patients with hearing problems is expected (WHO, 2021).

The Mongolian gerbil (*Meriones unguiculatus*) is a classic animal model for studying age-related hearing loss. It has a relatively short lifespan, and low-frequency hearing similar to that of humans (Ryan, 1976). It is easy to maintain (Cheal, 1986), and shows similarities to human age-related pathologies that are associated with hearing loss (reviews in Gates and Mills, 2005; Heeringa and Köppl, 2019). Gerbils are typically classified as “old” when they reach 36 months of age, which corresponds to 50% of their maximal lifespan potential, comparable to a human aged 61 years (reviewed by Castaño-González et al., 2024). Age-related morphological changes in the gerbil cochlea include loss of afferent synapses on inner hair cells (IHC; Gleich et al., 2016; Steenken et al., 2021; Bovee et al., 2024), loss of spiral ganglion neurons (Keithley et al., 1989; Suryadevara et al., 2001), hair-cell loss (Tarnowski et al., 1991; Adams and Schulte, 1997), and a lowered endocochlear potential (Schulte and Schmiedt, 1992; Schmiedt et al., 2002). Age-related changes to the efferent innervation of the gerbil cochlea have only been explored in a developmental context (Rontal and Echteler, 2003; Kaiser et al., 2011). Thus, even the efferent innervation in the young-adult gerbil is poorly characterized and its potential degeneration with advancing age remains unexplored.

Efferent fibers that innervate the cochlea originate in two spatially distinct subsets of neurons – medial olivocochlear (MOC) and lateral olivocochlear (LOC; review in Guinan, 2018) – although species-specific variations in their precise locations occur (reviewed in Brown, 2011; Lauer et al., 2022). The cell bodies of MOC neurons are located within the medial superior olivary complex and their myelinated axons innervate the outer hair cells (OHC) of either the contralateral or the ipsilateral ear, or rarely both (reviewed in Kimura and Wersall, 1962; Warr and Guinan, 1979; Guinan, 2011). In the mature ear, MOC neurons are primarily cholinergic and, to a lesser extent, use gamma-aminobutyric acid (reviews in Eybalin, 1993; Elgoyhen et al., 2019; Kitcher et al., 2022). The binding of acetylcholine promotes Ca^2+^ influx, which increases the opening of Ca^2+^-dependent K^+^ channels, and leads to hyperpolarization of OHC (Elgoyhen et al., 2019). Thus, MOC efferents exert cochlear gain control by reducing the voltage-driven electromotility of OHC (Brown and Nuttall, 1984; Cooper and Guinan, 2006). Furthermore, in young-adult mice, MOC fibers form *en passant* terminals on type-I auditory-nerve fibers within the inner spiral bundle (Hua et al., 2021), with as yet unknown function. MOC efferents may affect the progression of cochlear aging. Expression of a modified α9α10 cholinergic nicotinic postsynaptic receptor complex in OHC of mice, which provided enhanced olivocochlear inhibition, slowed the loss of spiral ganglion neurons and preserved hearing function (Boero et al., 2020). Conversely, lesioning olivocochlear neurons in the brainstem aggravated hearing loss and cochlear ribbon-synapse loss, although this was less clearly a specific MOC effect, and may have been compounded by LOC loss (Liberman et al., 2014).

LOC neurons of the second major efferent subsystem reside in and around the lateral superior olivary nucleus. The majority of LOC neurons innervates the ipsilateral ear (Warr and Guinan, 1979; Robertson, 1985). Their unmyelinated fibers cross the outer spiral lamina and synapse onto the unmyelinated processes of type-I afferents that run up toward and terminate on IHC (Warr and Guinan, 1979). While the majority of LOC neurons are cholinergic, they have been observed to also express other neurotransmitters and neuromodulators, including dopamine, calcitonin gene-related peptide, gamma-aminobutyric acid, glycine, and opioid peptides such as enkephalin (Eybalin, 1993; Kitcher et al., 2022). The function of the LOC system is more elusive due to the difficulties of directly assessing its physiological function (recent review in Lauer et al., 2022). However, the variety of neurotransmitters suggests functional variety. For example, dopamine is suggested to play a role in cochlear neuroprotection (Eybalin et al., 1993; D’Aldin et al., 1995; Oestreicher et al., 1997; Darrow et al., 2007). The LOC system may also regulate the spontaneous activity of type-I afferents (Ruel et al., 2006) and maintain the binaural balance needed for sound localization (Groff and Liberman, 2003; Darrow et al., 2006).

For the gerbil, an age-related loss of MOC and LOC neurons located in the brainstem, of 31 and 24%, respectively, has already been shown (Radtke-Schuller et al., 2015). Findings in human and mouse indicated species-specific changes in the peripheral terminals of aging olivocochlear efferents: In humans, LOC innervation density was unaffected, while MOC innervation was reduced with age (Liberman and Liberman, 2019). Age-related changes in CBA/CaJ mice yielded mixed results. While one study found a loss of innervation density for both MOC and LOC efferents (Grierson et al., 2022), a different study found even increased MOC innervation in apical regions, and mixed results for LOC cochlear innervation (Kobrina et al., 2020).

Here, we immunohistochemically labeled efferent terminals and hair cells in cochleae of young-adult and old gerbils. We quantified the signal at seven tonotopic locations along the organ of Corti and confirmed a typical pattern of LOC and MOC innervation density across the tonotopic axis. Both the presumed LOC and the presumed MOC innervation were significantly reduced in the cochleae of old gerbils. However, when correcting for the age-related losses in efferent target structures, such as loss in afferent fibers (Steenken et al., 2021) and OHC loss (Tarnowski et al., 1991; Adams and Schulte, 1997), the surviving cochlear circuitry appeared surprisingly intact.

## 2 Materials and methods

### 2.1 Animals

Results from a total of 26 cochleae from 20 Mongolian gerbils (*Meriones unguiculatus*) are reported here (Table 1). Young-adult gerbils (5 males, 7 females) were between 3 and 8 months old. Old gerbils (6 males, 2 females) were 36 to 41 months of age, corresponding to just over 50% of the species’ maximal lifespan potential (reviewed by Castaño-González et al., 2024). All experiments were in compliance with the relevant laws supervised by the Landesamt für Verbraucherschutz und Lebensmittelsicherheit (LAVES) of Lower Saxony. To minimize noise-induced hearing damage, animals lived their entire lives in a controlled, quiet environment with an average sound level of 48 and 55 dBA, outside and during working hours, respectively.

**Table 1:** Cochleae and cochlear positions examined in individual gerbils.

Animal ID	Side(s)	Age (months)	Sex	LOC, equivalent frequency locations	MOC, equivalent frequency locations
AHG61	R, L	41	m	0.5, 1, 2, 4, 8, 16, 32	0.5, 1, 2, 4, 8, 16, 32
AHG62	L	41	m	0.5, 1, 2, 4, 8, 16, 32	0.5, 8, 16
AHG64	R, L	36	m	0.5, 1, 2, 4, 8, 16, 32	2, 4, 8, 16, 32
AHG65	L	36	f	2, 8, 32	
AHG74	L	37	m	0.5, 1, 2, 4, 8, 16, 32	1, 2, 4, 8, 32
AHG75	R,L	36	f	0.5, 1, 2, 4, 8, 16, 32	1, 2, 4, 8, 16
BPG27	R	38	m	0.5, 1, 2, 4, 8, 16, 32	2, 8, 16
BPG36	R,L	36	m	0.5, 1, 2, 4, 8, 16, 32	1, 2, 16, 32
BPG11	R	4	m	1, 2, 4, 8, 16, 32	2, 8, 16
BPG16	L	8	m	0.5, 1, 4, 8, 16, 32	2, 8
BPG18	R,L	4	m	0.5, 1, 2, 4, 8, 16, 32	0.5, 1, 2, 4, 8, 16, 32
BPG19	L	3	m	0.5, 1, 2, 4, 8, 16, 32	0.5, 1, 2, 8, 32
BPG20	L	3	m	0.5, 1, 2, 4, 8, 16, 32	0.5, 1, 2, 4, 8, 16, 32
BPG21	R	6	f	0.5, 1, 2, 4, 8, 16, 32	0.5, 1, 2, 4, 8, 16, 32
BPG22	R,L	6	f	0.5, 1, 2, 4, 8, 16, 32	2, 4, 8, 32
BPG24	R	7	f	0.5, 1, 2, 4, 8, 16, 32	4, 8, 32
BPG31	R	5	f	0.5, 1, 2, 4, 8, 16, 32	0.5, 1, 2, 4, 8, 16
BPG32	R	6	f	1, 2, 4, 8, 16, 32	2, 4, 8, 16, 32
BPG33	R	6	f	0.5, 1, 2, 4, 8, 16, 32	0.5, 1, 2, 4, 8, 16, 32
BPG34	R	6	f	0.5, 1, 2, 4, 8, 16, 32	2, 4, 8, 16, 32

### 2.2 Tissue collection

Gerbils were euthanized with an overdose of sodium-pentobarbital (“Narcoren,” Merial GmbH, Hallbergmoos, Germany, 480 mg/kg body weight). When breathing stopped, gerbils were decapitated, and the bullae were rapidly dissected out of the head. The cochlea was accessed by breaking away the bone of the bulla, and the apex carefully opened by scratching the bone with fine forceps. The round-window membrane was perforated with the tip of the needle of an insulin syringe and the oval window opened by removing the stapes with forceps. Next, 450 μL 4% paraformaldehyde in phosphate buffered saline (PBS) was gently introduced through the oval window with an insulin syringe. Cochleae were post-fixed on ice in ice-cold fixative in falcon tubes, for 1 h, with brief manual agitation every 15 min.

### 2.3 Histology

Cochleae were decalcified in 0.5 M ethylenediaminetetraacetic acid for 2 days on a shaker at 8°C. After removing excess tissue, cochleae were permeabilized in 1% Triton X in PBS, on a shaker at room temperature for 1 h. Next, cochleae were washed in 0.2% Triton X in PBS 3 times for 5 min and subsequently incubated in blocking solution (3% bovine serum albumin in 0.2% Triton X PBS), for 1 h, on a shaker at room temperature. Primary antibodies included anti-synaptotagmin-1 (DSHB mAb48 (asv48)) IgG2b monoclonal mouse, Developmental Studies Hybridoma Bank, Iowa City, IA, USA; RRID: AB_2199314, diluted 1:200 (concentrate) or 1:20 (supernatant)) to label efferent terminals, and anti-MyosinVIIa (IgG polyclonal rabbit, Proteus Biosciences, Ramona, CA, USA; cat. No. 25e6790; RRID: AB_10015251; diluted 1:400) to label cochlear hair cells. Antibodies were diluted in blocking solution, and cochleae were incubated for 1 day at 37°C.

Cochleae were then washed 5 times for 5 min in 0.2% Triton X PBS. Secondary antibodies that specifically targeted the primary antibodies were then applied for 1 day at 37°C; these were AF488 goat anti-mouse (monoclonal secondary antibody IgG2b; Invitrogen, Thermo Fisher Scientific, Waltham, MA, USA; cat. no A-21141; RRID:AB_2535778; diluted 1:1000), and donkey anti-rabbit-AF647 (polyclonal secondary antibody; Life Technologies-Molecular Probes; cat. no. A-31573; RRID: AB_162544; diluted 1:1000). Cochleae were then washed twice for 5 min in 0.2% Triton X PBS, and 3 times for 5 min in PBS before they were micro-dissected into 6–10 pieces under a stereo microscope (Steenken et al., 2022). Pieces were mounted on slides using Vectashield Mounting Medium (Vector Laboratories, Burlingame, CA, USA, H-10 0 0).

### 2.4 Image acquisition

Images of cochlear pieces were acquired with a Nikon epi-fluorescence microscope system (Nikon Eclipse Ni-Ei with associated NIS Elements software, Version 4.30, 64-bit, Nikon, Minato, Tokyo, Japan). Cochlear length was assessed by measuring along the IHC row of each cochlear piece and specific cochlear locations that corresponded to selected frequencies (0.5 kHz, 1 kHz, 2 kHz, 4 kHz, 8 kHz, 16 kHz, 32 kHz) were identified relative to the length of the individual cochlea (Müller, 1996). High-resolution confocal stacks were acquired with a Leica TCS SP8 microscope system (Leica Microsystem CMS GmbH, Wetzlar, Germany) using an oil-immersion objective (40x, numerical aperture 1.3). Fluorescence tags were excited by different lasers [488-nm (Optically pumped semiconductor laser), and 638-nm (Diode)], and released photons were counted via a hybrid detector. Stacks were recorded in sequential scanning mode for the two channels, with a pixel resolution of *X,Y* = 63.1 nm and *Z* = 0.37 μm. Crosstalk between the channels was excluded with control specimens that were labeled with only one of the two antibodies. All confocal stacks were deconvolved (Huygens Essentials, Version 15.10, SVI, Hilversum, Netherlands) with default settings (maximum iteration: 80; signal to noise ratio: 10; quality threshold: 0.01), using a theoretical point-spread function.

### 2.5 Image analysis

All image analysis was carried out with ImageJ (FIJI, Schindelin et al., 2012). First, a defined region of interest (ROI) was cropped to only include either the IHC or OHC region. Thus, a median area of 7981.6 μm^2^ and containing 8.5–15.5 IHCs was analyzed for LOC terminals. Likewise, a median area of 6446.5 μm^2^ and containing 32–54 OHCs was analyzed for MOC terminals. Second, the fluorescent channels were separated. The channel with the MyoVIIa-label, displaying IHCs or OHCs, was used to manually count hair cells. The channel with the synaptotagmin label was used for all analyses of efferent innervation.

**Figure 1:**
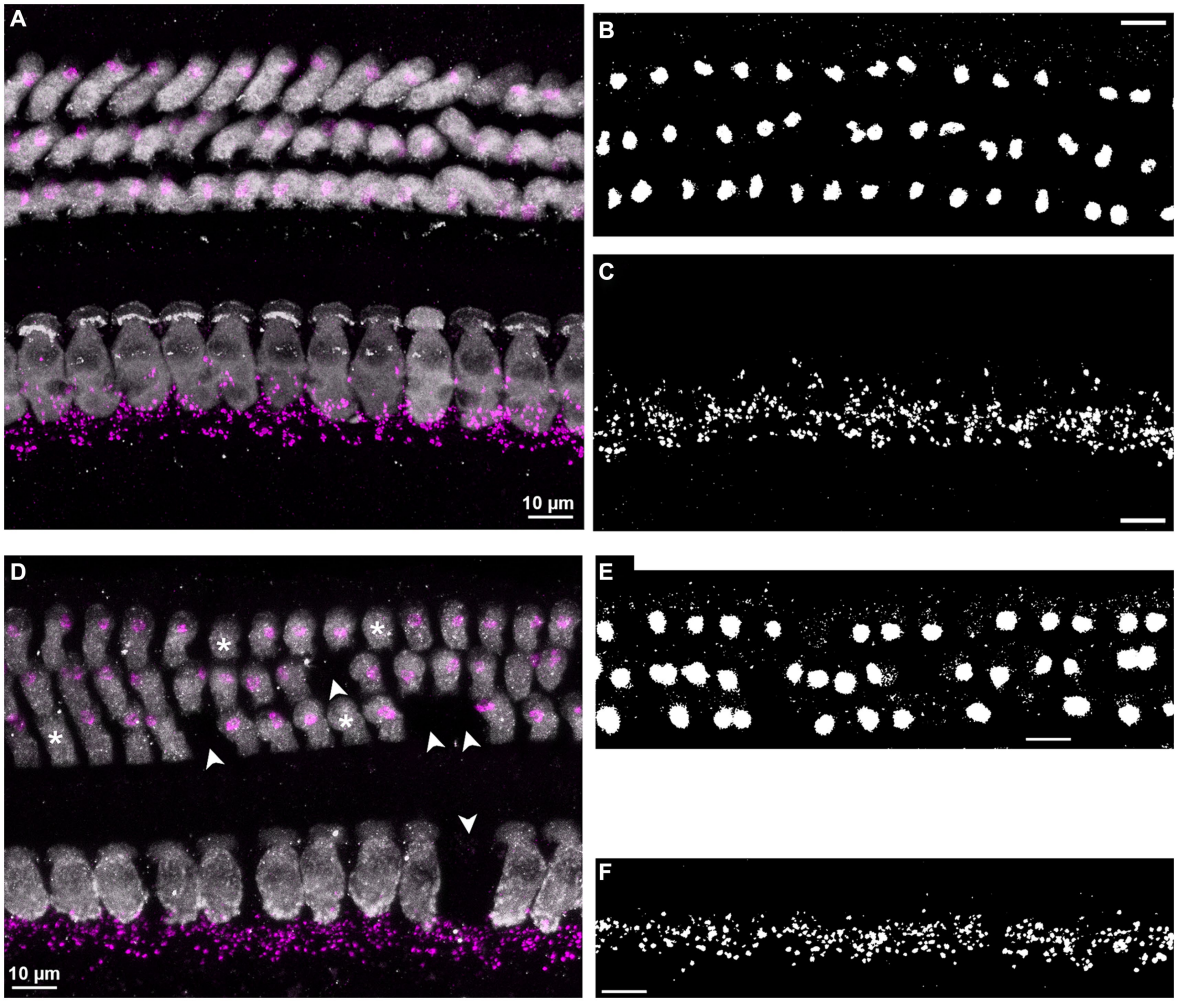
Examples of immunolabel and image analysis. **(A–C)** Illustrate an example from a young-adult female gerbil, aged 5 months, from a cochlear location corresponding to 8 kHz. **(D–F)** Illustrate an example from an old male, aged 41 months, from a cochlear location corresponding to 16 kHz. All scale bars correspond to 10 μm. **(A,D)** Confocal images, showing the entire organ of Corti, with IHCs (bottom) and OHCs (top). Hair cells (gray) and efferent terminals (magenta) were labeled with antibodies against myosin VIIa and synaptotagmin-1, respectively, and the synaptotagmin label was sharpened by deconvolution. The confocal stacks were collapsed into a 2D image by applying maximum intensity z-projection. Note that in the old individual **(D)**, several hair cells were missing (arrowheads) and 4 OHCs were orphaned, that is, were not associated with efferent terminals (asterisks). **(B,E)** The synaptotagmin channel only, after cropping the OHC area, applying average-intensity z-projection, and auto-thresholding with the algorithm “Li,” to binarize the labeled signal (presumably MOC synapses) within this region. **(C,F)** Same as in panels **(B,E)**, for the IHC area, but with maximum-intensity z-projection and auto-thresholding algorithm “moments.”

To quantify efferent innervation area, we followed a procedure according to Liberman and Liberman (2019). First, a z-projection using maximum (IHC region) or average (OHC region) pixel intensity was computed. The choice of average pixel intensity for the MOC analysis was governed by higher background levels in the OHC region, which was thus more effectively suppressed. After carefully comparing different auto-thresholding algorithms to best detect the labeled structures in these z-projections, we chose “Moments” for LOC, and “Li” for MOC. These were then applied to produce a binary image (examples shown in Figures 1B,C,E,F). Finally, the efferent innervation area was derived by multiplying the total ROI area with the fraction of synaptotagmin-labeled area within the ROI. For the OHC region, the area of synaptotagmin label was also determined separately for each OHC row. For this, particle detection (“particle analyzer” plugin in ImageJ) was applied and the areas of all detected particles in a given row of OHC were summed. Particles were only included if they were larger than 2 μm^2^, holes were filled, and objects on edges were included. Note that this procedure had been intended to yield a count of terminals which, however, was discarded as unreliable. Ultimately, only the sum of the detected particle areas was used. The result of this slightly modified procedure for measuring innervation area in individual OHC rows, when summed, was not significantly different from the total innervation area in the OHC region determined by the simpler procedure above (see Supplementary Figure S1) (mixed model ANOVA: *F*(1,170) = 0.92; *p* = 0.34). All area measurements were normalized per hair cell, by dividing total area by the HC count in the relevant ROI.

After adjusting brightness and contrast automatically, counts of individual MOC terminals were obtained manually, in 3D, from each image stack displaying both labeled channels. As for area, these counts were normalized per OHC by dividing the terminal count by the HC count in the relevant ROI. In addition, OHCs without terminals (orphaned OHCs), and terminals that were not associated with an OHC (orphaned terminals) were quantified separately.

Estimated counts of LOC terminals were also obtained in 3D by an automated procedure applied to the synaptotagmin signal of each image stack. First, the image stack was binarized by applying the auto-threshold filter “moments.” Next, the “3D objects counter” plugin in ImageJ, with a specified minimum object size of 100 voxels, was used to quantify LOC terminals. Objects at the edges were excluded. Here, too, the counts were normalized per IHC, by dividing the “objects counter” result by the HC count in the relevant ROI. The numbers of cochleae that were analyzed for each specific metric are listed in Table 2.

**Table 2:** Not every cochlear location, and every metric, could be evaluated in all 12 young-adult gerbils and 8 old gerbils.

Cochlear frequency	IHC (young/old)	OHC (young/old)	IHC innervation area (young/old)	IHC terminals (young/old)	OHC innervation area (young/old)	OHC terminals (young/old)
0.5	10/7	6/2	10/7	10/7	6/2	5/1
1	12/7	6/4	12/7	12/7	6/4	6/3
2	11/8	11/6	11/8	11/8	11/6	11/5
4	12/7	9/4	12/7	10/7	9/4	9/4
8	12/8	12/6	12/8	12/8	12/6	12/5
16	12/7	8/6	12/7	12/7	8/6	8/6
32	12/8	9/3	12/8	12/8	9/3	9/3

This table details the number of young-adult and old gerbils that contributed data, separately for each cochlear location (given in column 1), and each metric (columns 2–7): number of IHC, number of OHC, efferent innervation areas in proximity to IHC and OHC, and terminal numbers innervating the area around IHCs and OHCs.

### 2.6 Statistical analysis

Only one ear from each animal was initially analyzed. However, if a particular tonotopic location could not be evaluated, the same location was supplemented using if possible the other ear of the same individual. Reasons to exclude locations were: loss of HCs due to the dissection process, or abundant and unspecific background staining. In some cases, the structural integrity of OHC rows was compromised, thus that the analysis of total innervation area was feasible, but the allocation into distinct rows was impossible.

Statistical tests were carried out in Matlab (version 2022b, Mathworks, Natick, MA, USA) and IBM SPSS Statistics for Windows (version 29.0, IBM Corp, Armonk, NY, USA). Data from several tonotopic locations of individual animals do not represent independent samples. Therefore, linear mixed-model analyses of variance (ANOVA) were used to test for differences in efferent innervation, with age group and cochlear location as fixed factors. These were followed by post-hoc t-tests. Data describing findings in young-adult gerbils were tested with linear mixed-model ANOVAs and subsequent multiple comparisons (MATLAB function “multcompare” with default Tukey–Kramer correction).

## 3 Results

We used an antibody against synaptotagmin 1 to label efferent terminals. Synaptotagmin 1 is a calcium sensor attached to vesicles and as such controls fusion events to the presynaptic membrane (Wolfes and Dean, 2020). In mature C57Bl/6J mice, synaptotagmin 1 can be found in presynaptic efferent terminals on type-I fibers below IHCs (Reisinger et al., 2011; the OHC region was not investigated). In the gerbil cochlea, it labeled large structures terminating onto OHCs and smaller structures in close vicinity to the basal pole of IHCs (Figures 1A,D). This pattern is similar to that found below IHCs of C57BL6/J mice labeled with an antibody against synaptic vesicle protein 2 (Dondzillo et al., 2021), or at OHCs and below IHC in CBA/CaJ mice labeled with the same antibody (Kobrina et al., 2020). The same pattern was apparent below IHCs and at OHCs in CBA/CaJ mice labeled with an antibody against vesicular acetylcholine transporter (VAT; Grierson et al., 2022), as well as in the IHC and OHC area in the gerbil labeled with an antibody against synapsin (Barone et al., 2019). Based on these observations, we assume that anti-synaptotagmin comprehensively labeled all olivocochlear efferent presynaptic endings in our gerbils. It was, however, not possible to distinguish between LOC and MOC efferent terminals based on the anti-synaptotagmin label. Therefore for our analysis we classified all labeled structures in the OHC area as presumed MOC terminals and, likewise, all terminals within the IHC area as presumed LOC efferents, although we are aware that a minority of terminals innervating the IHC region might be formed by MOC fibers (Zachary and Fuchs, 2015; Hua et al., 2021).

### 3.1 Tonotopic variation of efferent innervation in young-adult gerbils

Since no quantitative data are available on cochlear efferent innervation in gerbils, we first established the typical pattern in young adults. Tonotopic variation of innervation was examined for seven locations along the cochlea, in total covering 75% of its length and equivalent best frequencies from 0.5 to 32 kHz (Müller, 1996). Two different metrics were obtained: (1) Area of synaptotagmin label in 2D projections, normalized per HC, and (2) numbers of terminals (OHC region, presumed MOC) or estimated numbers of terminals (IHC region, presumed LOC) counted in 3D, also normalized per HC.

**Figure 2:**
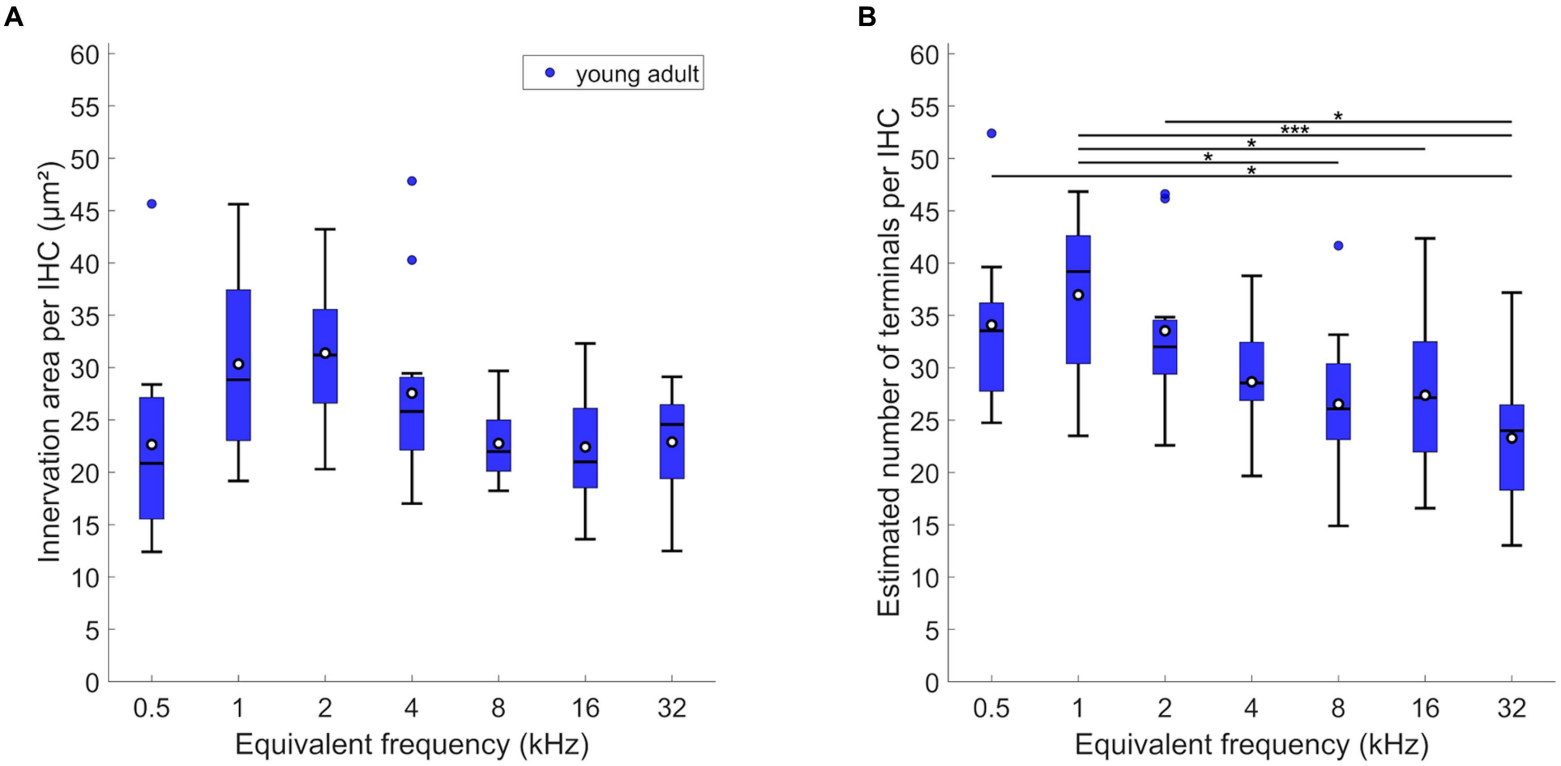
LOC innervation in young-adult gerbils. Data are displayed as **(A)** innervation area or **(B)** number of terminals per IHC. Box plots show the median (horizontal line), the 25th and 75th percentiles (upper and lower boundaries of the box), the data range without outliers (whiskers), and outliers (colored circles, defined as a value larger than three scaled median absolute deviations from the median). Means are also depicted, as white circles within each box. Note that not every cochlear location (expressed as equivalent frequency) included data from all 12 young-adult gerbils (see Tables 1, 2 for details). Asterisks denote significant differences between the cochlear frequencies connected by the respective line below (Tukey–Kramer-corrected post-hoc tests; *p < 0.05, ***p < 0.001).

LOC innervation area per IHC varied significantly along the length of the cochlea, with a peak at 1 to 2 kHz (Figure 2A; mixed-model ANOVA: *F*(6,74) = 3.52; *p* = 0.004). However, post-hoc Tukey–Kramer-corrected t-tests showed that this peak was not significantly different from all other locations. Thus, the LOC innervation area per IHC was nearly uniform along the entire cochlea. In contrast, the estimated number of terminals per IHC varied more clearly along the cochlea (Figure 2B; mixed-model ANOVA: *F*(6,72) = 5.37, *p* < 0.0005). The highest number of LOC terminals was found around and near IHC of the apical 1 kHz-location (mean: 36.96/median: 39.19 terminals per IHC). This number then decreased almost monotonically toward the most basal location evaluated (mean: 23.29/median: 24.0 terminals per IHC).

**Figure 3:**
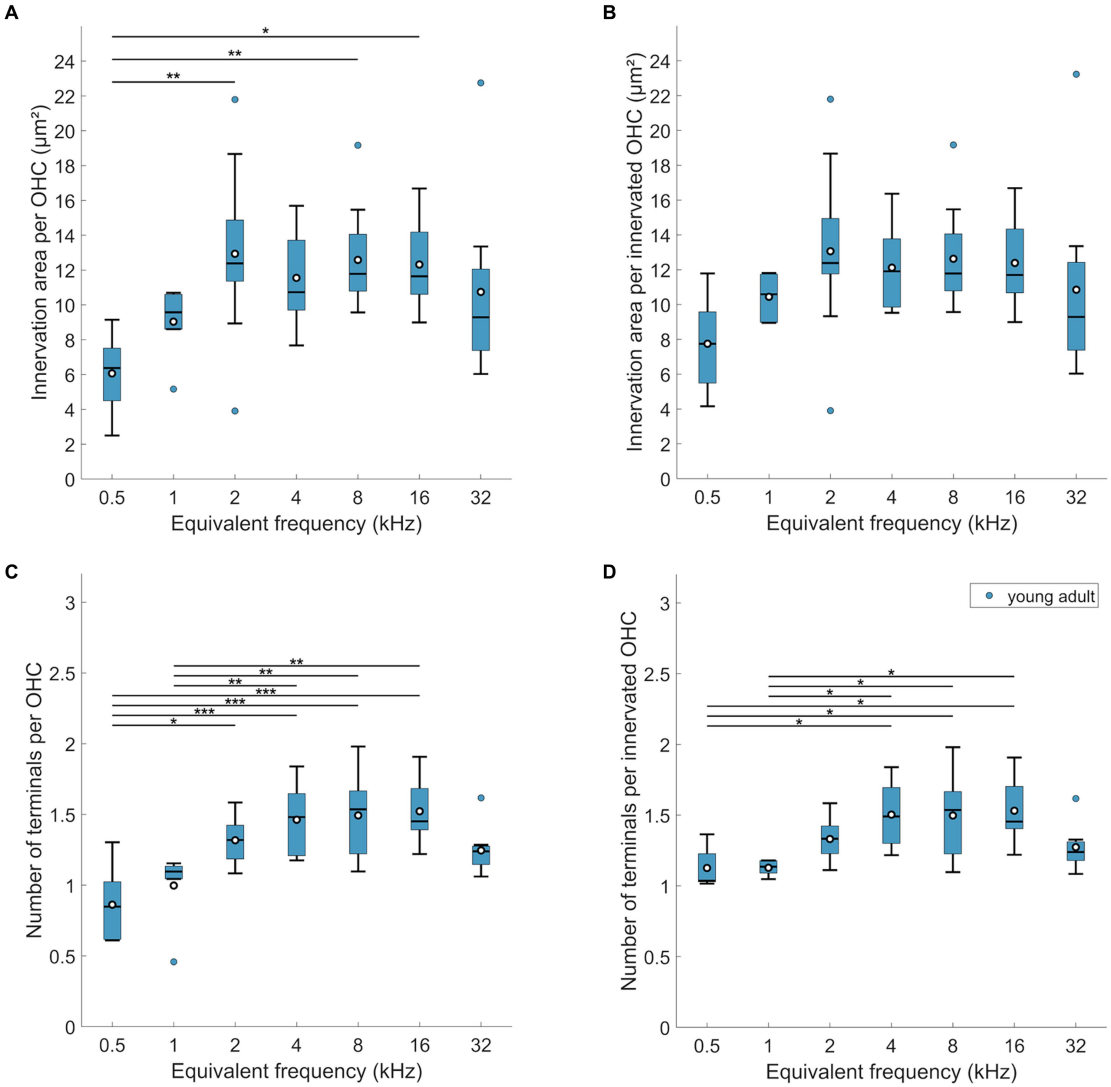
MOC innervation in young-adult gerbils. Data are displayed as **(A,B)** innervation area or **(C,D)** number of terminals, and were normalized either per OHC **(A,C)** or per innervated OHC, that is, corrected for orphaned OHCs **(B,D)**. Box plots show the median (horizontal line), the 25th and 75th percentiles (upper and lower boundaries of the box), the data range without outliers (whiskers), and outliers (colored circles). Means are also depicted, as white circles within each box. Note that not every cochlear frequency included data from all 12 young-adult gerbils (see Tables 1, 2). Asterisks denote significant differences between the cochlear frequencies connected by the respective line below (Tukey–Kramer-corrected post-hoc tests; *p < 0.05, **p < 0.01, ****p < 0.001).

MOC efferent innervation area per OHC varied significantly and was maximal at mid- to high-frequency, basal locations (Figure 3A; mixed-model ANOVA: *F*(6,54) = 3.48, *p* = 0.0055, and Tukey–Kramer corrected t-tests). At the extreme apex, the innervation area was smaller compared to most mid-frequency cochlear positions. The number of terminals per OHC was also differentially distributed along the cochlea (Figure 3C; mixed-model ANOVA: *F*(6,53) = 7.85, *p* < 0.0005). Very similar to innervation area, OHC at the extreme apex received the lowest numbers of efferent terminals, and the numbers peaked broadly at the mid-frequencies, between 2 and 16 kHz.

**Figure 4:**
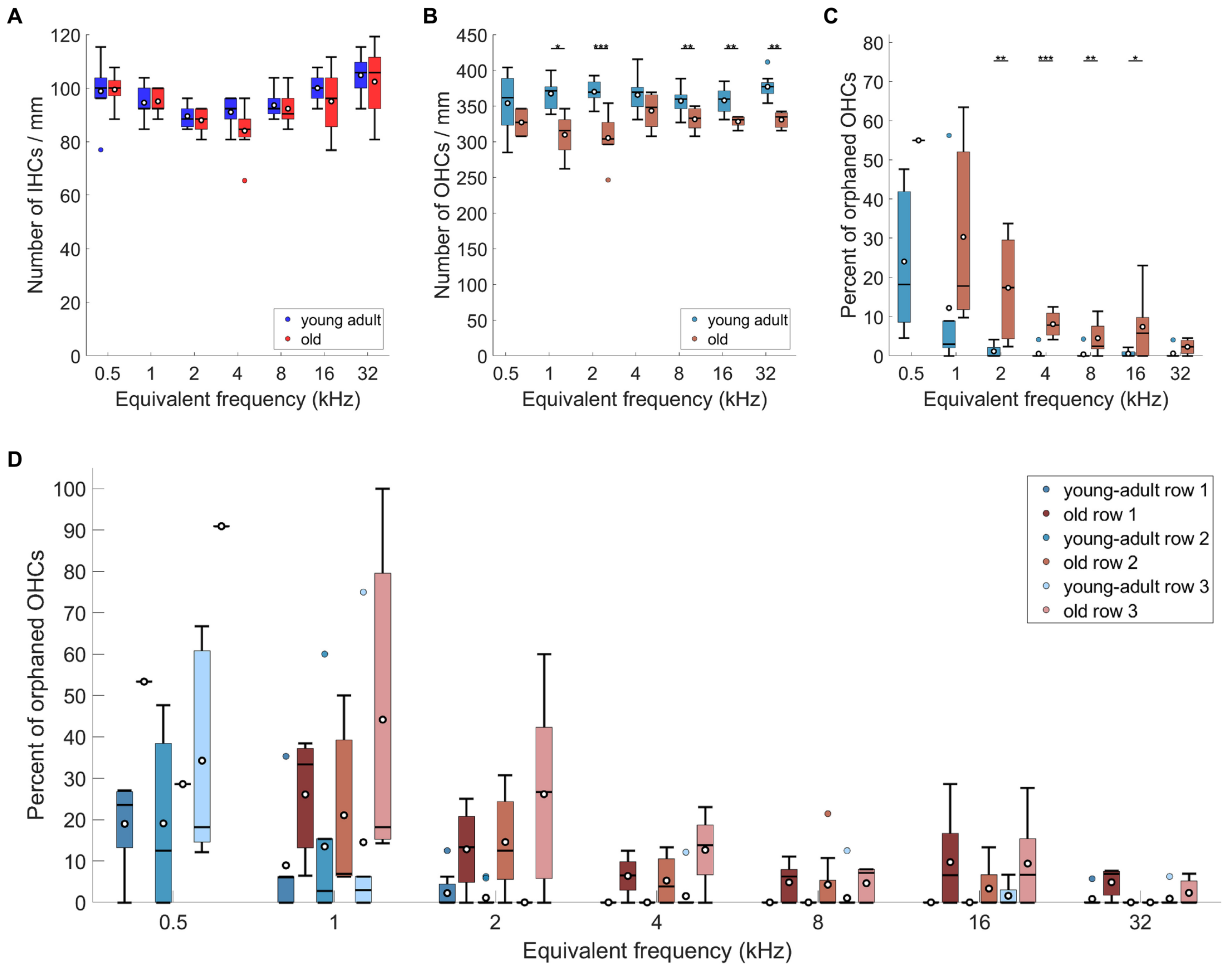
Age-related losses of hair cells and increase of orphaned OHCs. **(A)** Number of IHCs, **(B)** number of OHCs, both per mm of cochlear length, **(C)** percentage of non-innervated, i.e., orphaned OHCs, and **(D)** percentage of orphaned OHCs, separately for the three rows of OHCs. Box plots show the median (horizontal line), the 25th and 75th percentiles (upper and lower boundaries of the box), the data range without outliers (whiskers), and outliers (colored circles). Means are also depicted, as white circles within each box. Boxes in blue display data from young-adult gerbils, boxes in red represent old gerbils, and different shades of red and blue distinguish data from the IHC region, the OHC region, and the three rows of OHC, as indicated in the legends. Note that not every cochlear frequency included data for all 12 young-adult gerbils and 8 old gerbils (see Tables 1, 2). Asterisks denote significant differences between the age groups indicated by the respective line below (post-hoc tests; *p < 0.05, **p < 0.01, ***p < 0.001).

Interestingly, the mean below 1 at 0.5 kHz suggests that not all OHCs were efferently innervated at this location. Indeed, direct examination revealed orphaned OHCs in all young-adult cochleae at the extreme apex, less frequently at the 1 kHz location, and only very rarely at more basal locations (Figure 4C). Such orphans appeared randomly in all three rows of OHC. Figures 3B,D show revised versions of efferent innervation area / OHC and terminal number / OHC, where orphaned OHCs were ignored for the normalization. This eliminated any significant tonotopic variation for the labeled area / innervated OHC (Figure 3B; mixed-model-ANOVA: *F*(6,53) = 1.93, *p* = 0.0925) while the number of terminals per innervated OHC remained lower for apical cochlear locations compared to mid-cochlear locations (Figure 3D; mixed-model ANOVA: *F*(6,50) = 4.92, *p* = 0.0005).

Next, we explored whether the efferent innervation varied between the three rows of OHC, but found no differences. Neither the efferent innervation area, corrected for orphaned OHCs, (n-way-ANOVA: *F*(2,132) = 0,74, *p* = 0.48), nor the terminal number on innervated OHCs varied between the rows (n-way ANOVA: *F*(2,150) = 11.38, *p* = 0.39). Finally, we also screened for orphaned terminals, that is, terminals not apposed to an OHC. This was observed only once in young-adult cochleae.

### 3.2 Age-related loss of efferent target structures

The core interest of our research was focused on age-related changes of the efferent cochlear innervation that might indicate changes in efferent function. We thus aimed to differentiate between overall losses in efferent label and confounding losses of efferent target structures that are already known to occur in quiet-aged gerbils (Tarnowski et al., 1991; Steenken et al., 2021). To this end, the age-related loss of both IHCs and OHCs was quantified in our gerbil sample.

#### 3.2.1 Age-related loss of IHC and afferent fiber terminals

For IHC, no age-related loss was apparent (Figure 4A; n-way ANOVA: *F*(1,119) = 3,11, *p* = 0.08). The mean number of IHCs contained within the analyzed ROIs (representing 130 μm along the cochlear apex-base axis) was 12.49 (std + −1; median: 12.5) in young-adult gerbils and 12.19 (std + −1.34; median: 12) IHCs in old gerbils.

The main target of LOC efferents are not the IHC directly, but the type-I afferents coursing up to and connecting to the IHC. We did not investigate the age-related loss of afferents for the sample reported here. However, the loss of afferent synaptic connections was recently quantified for the same tonotopic locations in other samples from our gerbil colony (Steenken et al., 2021; Bovee et al., 2024). The implications of this for the novel results reported here will therefore be addressed in the discussion.

#### 3.2.2 Age-related loss of OHC and increase in orphaned OHC

Regarding OHC, young-adult gerbils had on average 47.34 (std + − 3.02; median: 48) OHCs contained within the analyzed ROIs (representing 130 μm along the apex-base axis). This number was significantly reduced in old gerbils (Figure 4B; n-way ANOVA: *F*(1,78) = 48.84, *p* < 0.0005), with only 42.11 (std + −3.39; median: 42.5) OHCs contained in the same-sized ROIs. On average, this represents a fairly small loss of 11% OHC. Post-hoc tests at specific cochlear locations revealed that the age-related loss was significant for 5 of the 7 locations evaluated, sparing only the extreme apex (0.5 kHz) and a mid-cochlear region (4 kHz).

Since young-adult gerbils typically displayed a minor fraction of orphaned OHCs (devoid of efferent innervation) in the cochlear apex, we explored whether this phenomenon increased and/or spread over larger cochlear regions with age. Compared to young-adult gerbils, old gerbils showed an overall significant increase in the percentage of orphaned OHCs (Figure 4C; n-way ANOVA: *F*(1,70) = 21.76, *p* < 0.0005). Frequency also had a significant effect (n-way ANOVA: *F*(6,70) = 10.07, *p* < 0.0005), confirming larger numbers of orphaned OHCs in the apex; these decreased toward the base. When comparing the numbers at specific cochlear locations (post-hoc tests), an age-related increase was significant for mid-cochlear frequencies (2–16 kHz), suggesting a spread of the phenomenon toward more basal locations in old cochleae (Figure 4C). An increase in orphans was observed for all three rows of OHC (Figure 4D), with a significant overall difference between the rows (n-way ANOVA: *F*(2,222) = 8.92, *p* = 0.0002). Posthoc Tukey–Kramer-corrected t-tests, however, did not confirm significant differences between specific rows. The large significant effect in the ANOVA model appeared to be dominated by the very small data set (*n* = 1) obtained for old gerbils at the cochlear location corresponding to 0.5 kHz. When this location was omitted in the ANOVA model, the difference became less significant (n-way ANOVA: *F*(2,208) = 3.56, *p* = 0.03). In both ANOVAs, no interaction was apparent between rows and age or frequency. Finally, orphaned terminals (without an OHC close by) were occasionally observed, but remained rare (a total of 6 across all cochlear locations evaluated in all gerbils, of which 5 were found in old cochleae).

### 3.3 Age-related changes of efferents in IHC region (presumed LOC)

**Figure 5:**
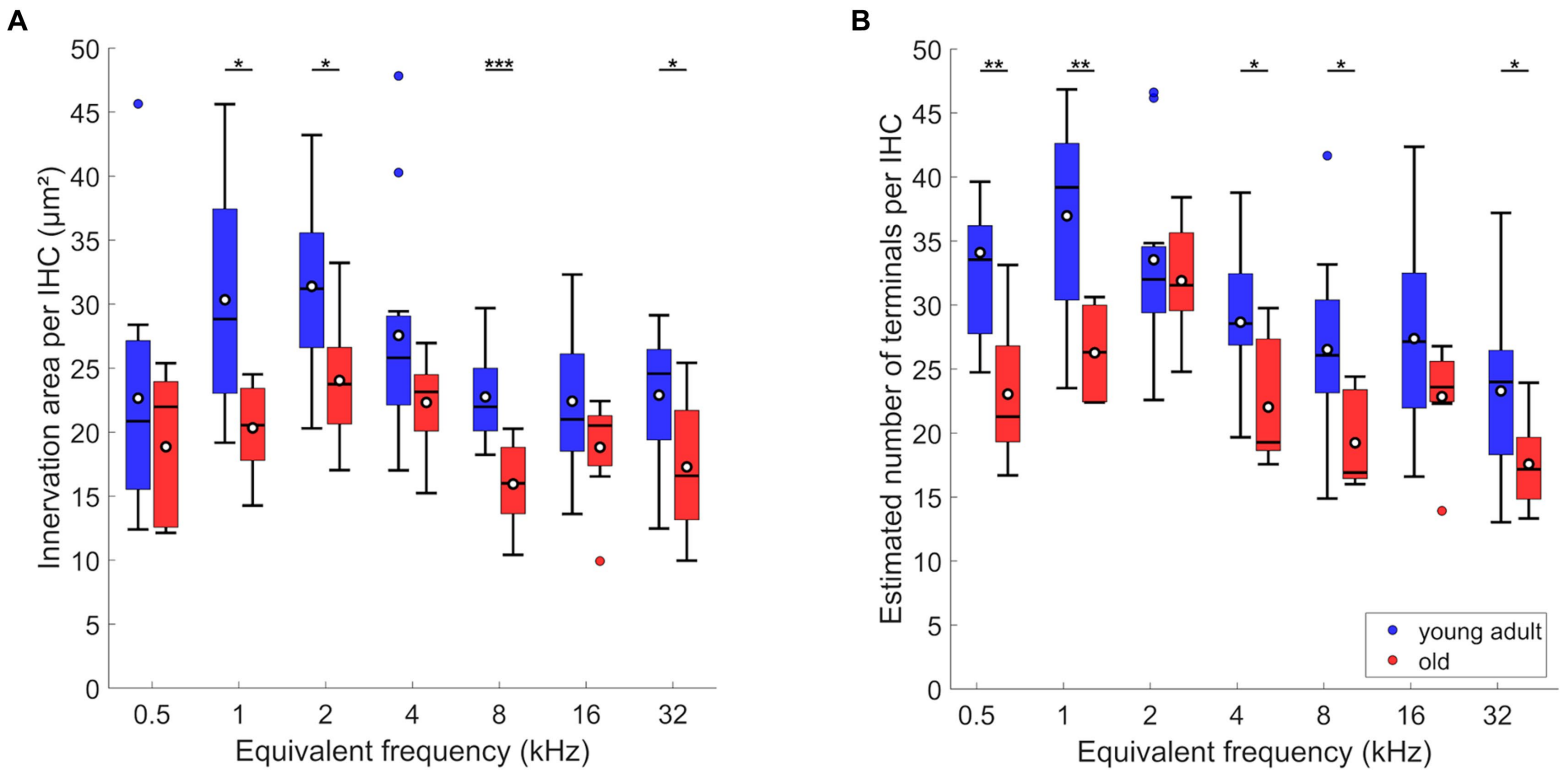
Age-related changes of efferents in IHC region. **(A)** Innervation area and **(B)** number of terminals per IHC. Box plots show the median (horizontal line), the 25th and 75th percentiles (upper and lower boundaries of the box), the data range without outliers (whiskers), and outliers (colored circles). Means are also depicted, as white circles within each box. Boxes in blue display data from young-adult gerbils, boxes in red represent old gerbils. Note that not every cochlear frequency included data for all 12 young-adult gerbils and 8 old gerbils (see Tables 1, 2). Asterisks denote significant differences between the age groups indicated by the respective line below (post-hoc tests; *p < 0.05, **p < 0.01, ***p < 0.001).

In comparison to young adults, old gerbils showed a significant decrease in the LOC innervation area per IHC (Figure 5A; n-way ANOVA: *F*(1,119) = 29.09; *p* < 0.0005). Over all tonotopic locations, the age-related loss amounted to 24%. As already seen for young adults alone, there was a significant effect of frequency (n-way ANOVA: *F*(6,119) = 4.87, *p* < 0.0005), suggesting tonotopic variation in LOC innervation. However, no interaction was found between frequency and age, indicating similar tonotopic variation in young-adult and old gerbils, and a uniform loss of innervation area along the aging cochlea. Post-hoc comparisons confirmed significant age-related losses for 4 of the 7 cochlear locations evaluated (Figure 5A).

Compared to young adults, old animals also showed significantly fewer efferent terminals per IHC (Figure 5B; n-way ANOVA: *F*(1,116) = 35.81, *p* < 0.0005). Furthermore, an effect of frequency was confirmed (n-way ANOVA: *F*(6,116) = 9.26, *p* < 0.0005), but there was no significant interaction between age and frequency, suggesting a uniform loss of terminals per IHC along the cochlea. Over all tonotopic locations, the age-related loss amounted to 23%. Post-hoc comparisons revealed that the age-related loss was significant for most cochlear locations (5 out of 7 evaluated; Figure 5B).

In summary, old gerbils had lost around 23% of their presumed LOC innervation. This loss appeared nearly uniform along the tonotopic axis. The two metrics evaluated here, labeled area and estimated number of terminals, yielded nearly identical results.

### 3.4 Age-related changes of efferents in the OHC region (presumed MOC)

**Figure 6:**
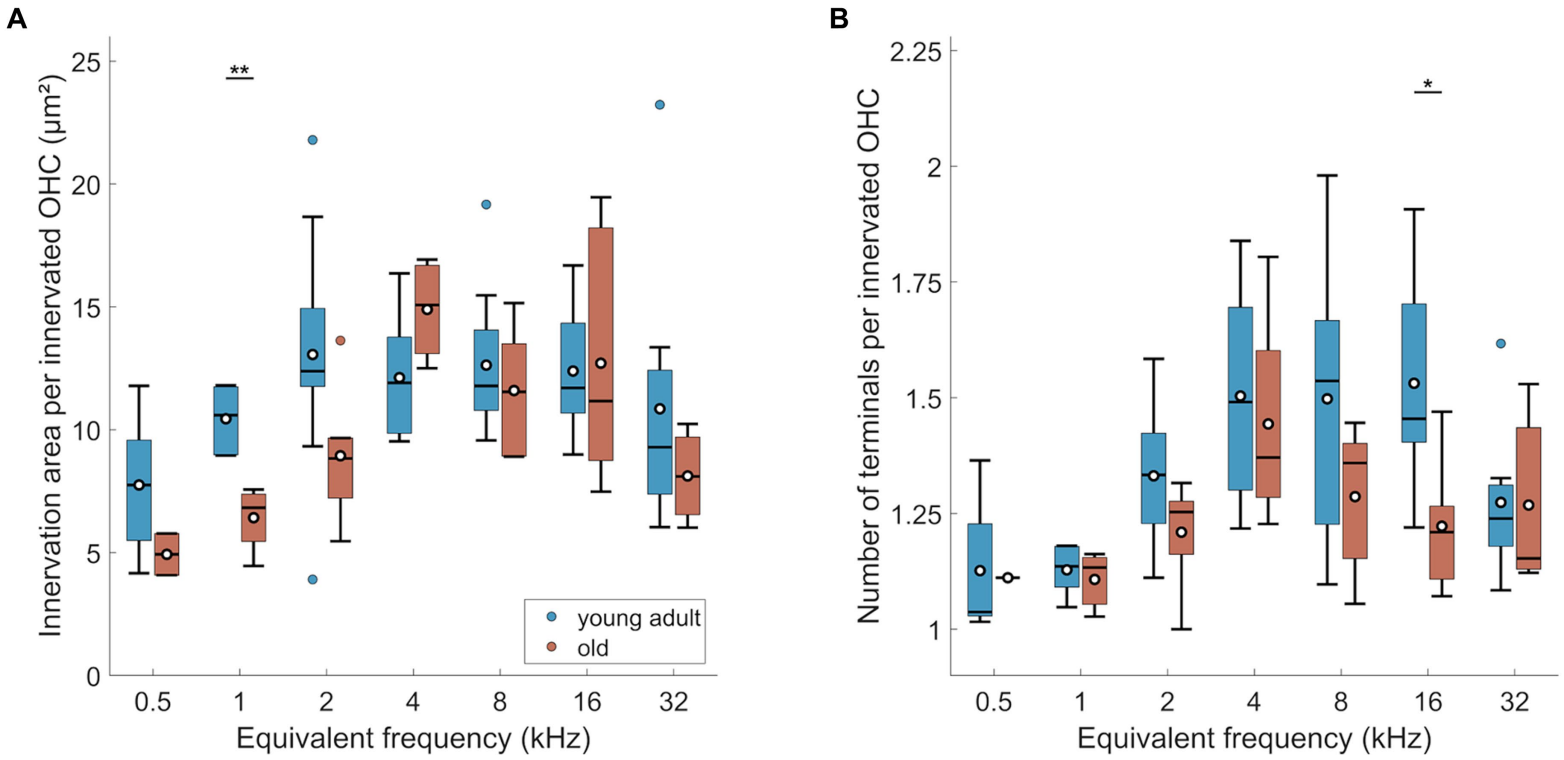
Age-related changes of efferents in OHC region. **(A)** Innervation area and **(B)** number of terminals per innervated OHC. Box plots show the median (horizontal line), the 25th and 75th percentiles (upper and lower boundaries of the box), the data range without outliers (whiskers), and outliers (colored circles). Means are also depicted, as white circles within each box. Boxes in blue display data from young-adult gerbils, boxes in red represent old gerbils. Note that not every cochlear frequency included data for all 12 young-adult gerbils and 8 old gerbils (see Tables 1, 2). Asterisks denote significant differences between the age groups indicated by the respective line below (post-hoc tests; *p < 0.05, **p < 0.01, ***p < 0.001).

The MOC innervation area per OHC, corrected for orphaned OHCs, showed a significant age effect (Figure 6A; n-way ANOVA: *F*(1,77) = 4.4, *p* = 0.0392). Over all tonotopic locations, the age-related loss amounted to 12%. However, post-hoc comparisons for each cochlear location revealed that only the 1 kHz-location showed a significant age-related reduction of MOC innervation area per OHC. Frequency also had a significant effect (n-way ANOVA: *F*(6,77) = 4.83, *p* < 0.0005), but there was no significant interaction between age and frequency. Thus, the tendency toward smaller innervation area in the apex, already observed in young adults, remained unchanged in old gerbils (Figure 3B).

Manual counts of terminals per innervated OHC revealed a significant overall loss of terminal number in old gerbils (Figure 6B; n-way ANOVA: *F*(1,70) = 4.25, *p* = 0.043). Over all tonotopic locations, this age-related loss amounted to 9%. However, similar to innervation area, most cochlear locations did not reveal significant age-related losses with location-specific post-hoc tests. Here, only the 16 kHz-location showed a significant decline in the number of terminals (Figure 6B). As already shown for young-adult gerbils alone, the number of terminals per OHC was also frequency dependent (n-way ANOVA: *F*(6,70) = 3.8, *p* = 0.0025), with lower numbers of terminals per innervated OHC in the apex. An interaction between age and frequency was absent, indicating a trend toward loss of terminal numbers all along the cochlea.

**Figure 7:**
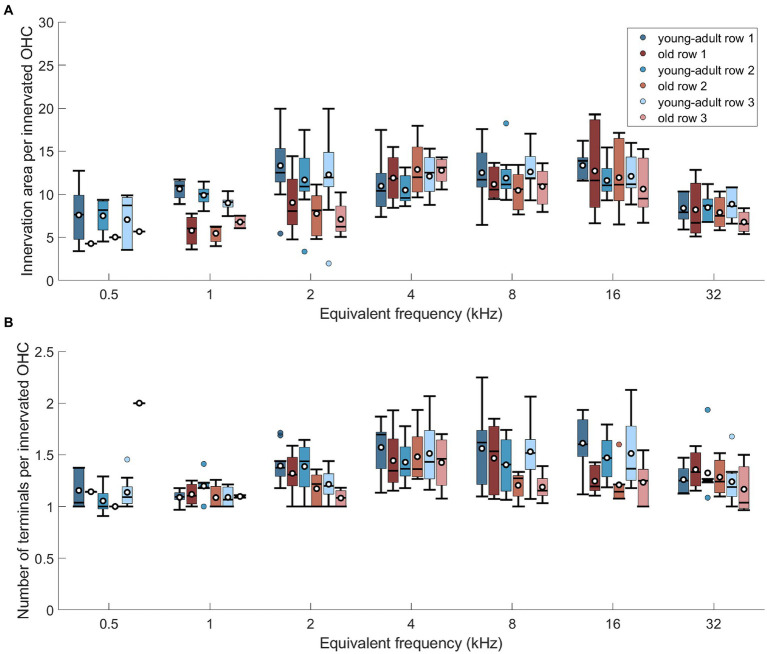
No differential, age-related changes of efferents across the 3 rows of OHCs. **(A)** Innervation area per innervated OHC at seven cochlear frequencies displayed separately for the three rows of OHC, for both age groups. **(B)** Same as in panel **(A)**, but for number of terminals per innervated OHC. Box plots show the median (horizontal line), the 25th and 75th percentiles (upper and lower boundaries of the box), the data range without outliers (whiskers), and the outliers (colored circles). Means are also depicted, as white circles within each box. Boxes in blue display data from young-adult gerbils, boxes in red represent old gerbils, and different shades distinguish the three rows of OHC. Note that not every cochlear frequency included data for all 12 young-adult gerbils and 8 old gerbils (see Tables 1, 2). Post-hoc tests revealed no significant differences between the rows at any cochlear frequency.

To explore whether the three OHC rows are differentially affected by age, the efferent innervation area (Figure 7A) and the number of terminals per OHC (Figure 7B) were assessed separately for each row. Innervation area was not affected by row number (Figure 7A; n-way-ANOVA: *F*(2,206) = 0.66, *p* = 0.516). Likewise, terminal number per OHC was not different between the rows (Figure 7B; n-way-ANOVA: *F*(2,221) = 1.6, *p* = 0.204).

In summary, we observed a tendency for mild losses in MOC innervation of surviving OHC, of around 10%. There was no evidence for differential losses along the tonotopic axis or between the three rows of OHC. Thus, the typical mid- to high-frequency bias of MOC innervation density was retained in old gerbils. Both metrics that were evaluated here, labeled area and number of terminals, agreed in their principal results.

## 4 Discussion

The present study quantified the olivocochlear innervation at seven tonotopic locations along the organ of Corti of gerbils, with the primary aim to probe for age-related changes between young adults and aged individuals. We found that innervation area and number of terminals of presumed LOC cochlear efferents were significantly reduced in the cochleae of old gerbils. This loss might be confounded by an equivalent loss in afferent fibers (Steenken et al., 2021), such that the surviving afferents retain a near-normal efferent innervation. Contrary to IHC, an age-related OHC loss was found, as previously reported (Tarnowski et al., 1991; Adams and Schulte, 1997). Furthermore, an increased number of non-efferently innervated OHCs, i.e., orphaned OHCs, was apparent in old gerbils. The loss of putative MOC efferents on surviving and still innervated OHCs was mild but significant. We found no difference in efferent innervation between the three rows of OHCs, and no differential age-related loss.

### 4.1 Efferent innervation patterns in young adults

In the young-adult gerbil, the presumed LOC innervation in the IHC region showed a tendency to peak apically, around 1–2 kHz equivalent best frequency, and decrease nearly monotonically toward the cochlear base. The presumed MOC innervation of OHCs tended to peak broadly and more basally, in regions between 2 and 16 kHz equivalent best frequency. These tonotopic patterns appear to be quite typical across species.

LOC innervation density tends to show a bias toward the cochlear apex, if any. An apical bias was observed in rat (Vetter et al., 1991) and human (Liberman and Liberman, 2019) and, less pronounced, in cat (Liberman et al., 1990) and gerbil (this study). In young-adult CBA/CaJ mice, LOC innervation is nearly uniform along the tonotopic gradient (Maison et al., 2003; Kobrina et al., 2020; Grierson et al., 2022). In the brainstem of gerbils, both Kaiser et al. (2011) and Radtke-Schuller et al. (2015) found more LOC neurons in the medial, high-frequency limb of the lateral superior olive that houses the LOC neurons that innervate basal, high-frequency regions of the cochlea (Sanes et al., 1989). This apparent disagreement with our cochlear data, where the efferent innervation of IHCs tended to be significantly denser in apical cochlear regions (Figure 2), suggests a more extensive branching pattern of LOC peripheral axons in the cochlear apex.

MOC innervation tends to peak broadly in mid-cochlear regions, which then also coincides broadly with the range of most sensitive hearing in the behavioral audiogram of a given species, as previously shown for cat (Fay, 1988; Liberman et al., 1990), human (Zwicker and Fastl, 1990; Liberman and Liberman, 2019), CBA/CaJ mouse (Maison et al., 2003; Radziwon et al., 2009; Grierson et al., 2022) and guinea pig (Heffner et al., 1971; Kujawa and Liberman, 1997; Liberman and Liberman, 2019). This was confirmed here for the gerbil, whose behavioral sensitivity is best between 1 and 20 kHz (Ryan, 1976). The number of presumed MOC terminals on gerbil OHC – between 1 and 2 – is similar to that shown for CBA/CaJ mice, also using immunolabeling (Maison et al., 2003; Grierson et al., 2022), but appears low compared to the counts from electron-microscopic reconstructions in cat (0–9; Liberman et al., 1990) and guinea pig (4–13; Hashimoto and Kimura, 1988). Mouse and gerbil are also the only species where no trend of decreasing MOC innervation area across the OHC rows was evident (Maison et al., 2003; this study). In the cat, a decrease in both efferent area and terminal number from OHC row 1 to row 3 was quantified (Liberman et al., 1990), and similar qualitative observations were reported for guinea pig, rhesus monkey, and human (Hashimoto and Kimura, 1988; Liberman and Liberman, 2019). Together, this suggests that compared to the other species investigated so far there are fewer MOC terminals on individual OHCs of gerbil and mouse, but an even distribution among the OHC rows.

A perhaps surprising finding in our study was that even in young-adult gerbils, orphaned OHC with no efferent terminals were regularly seen in the cochlear apex. The phenomenon of orphaned OHC has also been reported (but not quantified) for young-adult rhesus monkeys and humans, where it was associated with a general trend of decreasing MOC innervation density from row 1 to row 3 (Liberman and Liberman, 2019).

### 4.2 Age-related changes to bulk efferent innervation in IHC region (presumed LOC)

For two different metrics of efferent innervation, we showed a significant and nearly uniform loss of about 23% in the IHC region of old gerbils. This agrees remarkably well with counts of presumed LOC neurons in and around the lateral superior olive in the brainstem of gerbils (Radtke-Schuller et al., 2015). There, an overall loss of 24% was found in gerbils that were of similar old age to those in the present study (2.5–3.5 years), compared to young adults. The loss of LOC neurons was disproportionally higher in the medial, high-frequency limb of the LSO (Radtke-Schuller et al., 2015). This does not agree with our observation of a uniform loss across the tonotopic axis. However, subtle differences between the gerbil samples (our gerbils were raised in a controlled, quiet environment), or as yet unknown differences in the cochlear branching patterns of LOC axons in the cochlear apex versus the base (as suggested above, see section 4.1), could conceivably account for this difference.

Similar, extensive reductions in bulk metrics of IHC efferent innervation have not so far been observed in aging individuals of other species. An exception are C57BL/6 mice (Stamataki et al., 2006; Lauer et al., 2012; Jeng et al., 2021), a strain that is well known for its accelerated auditory aging phenotype (e.g., Spongr et al., 1997) and thus difficult to compare across species. For comparing equivalent ages across species, we refer here to their respective maximum lifespan potential (reviewed in Castaño-González et al., 2024). In humans, no significant change was found in LOC innervation, quantified as ChAT-immunolabeled area per IHC (very similar to one of our metrics in gerbil). This was in individuals up to 86 years (Liberman and Liberman, 2019), equivalent to 70% of the human maximal lifespan potential, that is, even beyond the lifespan range explored here for gerbils. Aging CBA/CaJ mice showed no consistent changes in immunolabeled area per IHC (Kobrina et al., 2020) or only losses limited to a narrow frequency range around 32 kHz, for immunolabeled volume (Grierson et al., 2022). This agreed with only minor losses, of 13%, in the overall numbers of LOC brainstem neurons (Vicencio-Jimenez et al., 2021). The mice evaluated in those studies were between 18 and 30 months old, equivalent to about 50–80% of their maximal lifespan potential.

We note that the efferent innervation metrics compared above were all normalized per surviving IHC. Age-related loss of IHC is typically minor, especially in individuals that lived in a controlled, quiet environment. However, a controlled environment is not always feasible. The extent of age-related IHC loss varies between studies, and is often region-specific within the cochlea (e.g., human: Liberman and Liberman, 2019; Wu et al., 2019; CBA/CaJ mice: Spongr et al., 1997; Sergeyenko et al., 2013; Parthasarathy and Kujawa, 2018; Kobrina et al., 2020; Grierson et al., 2022). This should therefore be controlled for when comparing innervation metrics across studies. In our sample, the number of IHCs was not significantly different between young-adult and old gerbils (Figure 4A), in agreement with previous observations in quiet-aged gerbils of similar age (Tarnowski et al., 1991; Adams and Schulte, 1997).

### 4.3 Confounding age-related loss of type-I afferents

**Figure 8:**
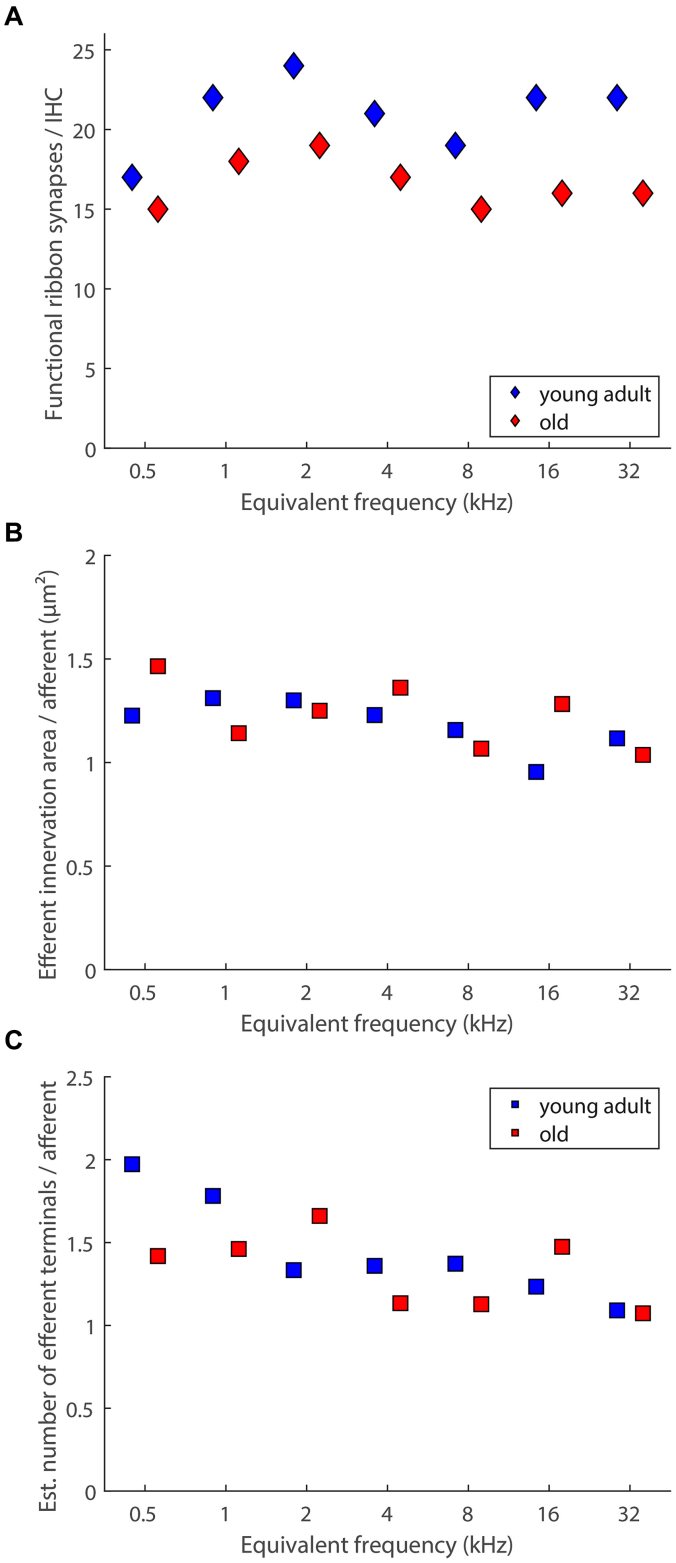
Age-related loss of presumed LOC innervation explained by loss of its primary target (afferent innervation). **(A)** Medians of functional ribbon synapses per IHC (replotted from Steenken et al., 2021, their Figure 3). **(B)** An estimate of efferent innervation area per afferent dendrite connecting to IHCs, derived by dividing the median values shown in Figure 5A by the data in panel **(A)**. **(C)** An estimate of the number of efferent terminals on each afferent dendrite connecting to IHCs, derived by dividing the median values shown in Figure 5B by the data in panel **(A)**.

An important confounding factor that was not addressed in the current sample, and not in any of the studies discussed above (see section 4.2), is the concomitant loss of type-I afferents, the primary target of LOC efferent innervation. For gerbils, the loss of afferent synaptic connections, assessed as co-localized puncta immunolabeled for pre-synaptic ribbons and post-synaptic glutamate receptor patches, was recently quantified, for the same 7 tonotopic locations, in another sample from our colony, and for the same age groups (Steenken et al., 2021). Compared to young adults, old gerbils showed significant losses of afferent synapses, of, on average, around 20% (Steenken et al., 2021). Medians of these published data, for each tonotopic location, are re-plotted in Figure 8A. It is well established that type-I afferent fibers very rarely branch and typically form only a single synapse with a single IHC (reviewed in Moser et al., 2006). Thus, a loss of synaptic connections means an equivalent loss of afferent fibers in the immediate proximity of IHC. Using this assumption, Figures 8B,C show estimates of the efferent innervation per afferent fiber under each IHC, derived by dividing the median values shown in Figures 5A,B by the medians shown in Figure 8A. A formal statistical test is not feasible, because the quantification of afferent and efferent innervation derived from different individuals. However, the age-related differences in Figures 8B,C appear to be negligible.

For aging gerbils, we thus conclude that LOC neurons and their peripheral terminals in the cochlea degenerate together with their principal targets, the type-I afferent peripheral dendrites. This suggests that the efferent innervation of surviving targets in the IHC region of aging gerbils is still intact – at least numerically. More subtle changes, such as the intriguing shift toward a higher proportion of axosomatic efferent synapses on aging IHCs that was observed in C57BL/6J mice (Lauer et al., 2012; Zachary and Fuchs, 2015; Jeng et al., 2021) remain possible, and should be explored in future studies. Of note, the small efferent terminals in the IHC area are notoriously difficult to resolve individually in immunolabeled material. The absolute numbers of presumed LOC terminals that were estimated in the present study are thus almost certainly underestimates. High-resolution electron microscopic data from young-adult cat (Liberman et al., 1990) and mouse (Hua et al., 2021) have shown typical numbers of 5–20 efferent terminals on one afferent terminal dendrite.

As discussed above (see section 4.2), in humans and CBA/CaJ mice, there is little evidence for age-related degeneration of LOC neurons and their peripheral terminals. This is despite drastic age-related losses in afferent synapses or peripheral dendrites to the IHCs. For CBA/CaJ mice of comparably old ages to those evaluated for LOC bulk innervation, a mean loss of between 20 and 50% of afferent synapses (depending on cochlear location and precise age) has been shown (Sergeyenko et al., 2013; Kobrina et al., 2020). In humans, the age-related loss of peripheral afferent dendrites was even more drastic, at 45–65%, again depending on cochlear location and precise age (Wu et al., 2019). Thus, in mice and humans, the loss of afferent targets does not appear to result in an equivalent loss of LOC innervation, in contrast to our observations in the gerbil. It remains to be clarified whether these species-specific differences might be explained by different time courses of afferent retraction and degeneration. Degeneration of the spiral ganglion cell body occurs significantly later than the detachment of the peripheral dendrite from the IHC ribbon synapse, such that counts at different locations yield significantly different results for the same chronological age (Makary et al., 2011; Sergeyenko et al., 2013; Wu et al., 2019). Thus, if the retraction of the primary dendrite happened relatively more quickly in gerbil, a delay between observed afferent synapse loss and observed LOC loss might be less noticeable. Interestingly, a spatial re-distribution of presumed LOC terminals occurred in aging CBA/CaJ mice, such that many shifted to a position further below the IHC’s basal pole (Grierson et al., 2022), which could reflect afferent dendrites retracting together with the efferent terminals attached to them. A minority was also seen further up on the IHC than typically observed in young adults (Grierson et al., 2022), which could reflect the exploration of new targets by orphaned LOC branches, and possibly axosomatic re-connections (see also section 4.5). Higher-resolution ultrastructural studies are needed to resolve the dynamics of potential efferent retraction and re-distribution that are associated with age-related changes to their targets.

### 4.4 Age-related changes to efferent innervation in OHC region (presumed MOC)

The overall density of MOC terminals per OHC declined only mildly in old gerbils, by about 10%, and was not consistently significant for most cochlear locations (Figure 6). Comparisons with previous studies are problematic in detail and highlight that for evaluating MOC innervation in particular, it is crucial to be aware of subtle methodological differences when comparing species or ages. Furthermore, C57BL/6 mice, with their accelerated auditory aging phenotype, are again a special case (Fu et al., 2010; Jeng et al., 2020) and are excluded from our species comparison. Two separate confounding factors need to be considered. First, OHC loss in aged cochleae tends to be more severe than IHC loss, and more variable between species and studies, even for comparable lifespan stages. The loss of OHCs in our quiet-aged gerbils was, on average, a moderate 11%, which is consistent with previous gerbil studies, despite slightly different tonotopic patterns of this loss (Tarnowski et al., 1991; Adams and Schulte, 1997). In humans and mice, more drastic OHC losses were observed that were typically most pronounced at the apical and basal extremes of the cochlea (Spongr et al., 1997; Sergeyenko et al., 2013; Liberman and Liberman, 2019; Kobrina et al., 2020; Wu et al., 2020; Grierson et al., 2022). If a specific evaluation of the efferent innervation status is aimed for, it is thus imperative to normalize MOC innervation metrics to the number of surviving OHCs. After doing so, age-related declines are typically moderate or cannot be clearly demonstrated. In humans of comparably old ages to the gerbils of the present study, VAT-immunolabeled area per surviving OHC was not significantly different to younger adults, but it did significantly decline in even older individuals, aged up to 70% of the human maximum lifespan potential (Liberman and Liberman, 2019). In C3H/HeJ mice (a strain with a similar auditory-aging phenotype to CBA/CaJ), no loss in the number of efferent synapses per surviving OHC was evident (Jeng et al., 2020), but the mice were still comparatively young, only up to 40% of their maximum lifespan potential. In CBA/CaJ mice of much more advanced ages, up to 80% of their maximum lifespan potential, the loss of VAT-immunolabeled area per surviving OHC was borderline significant when compared to young adults (Grierson et al., 2022).

Furthermore, conclusions may differ, depending on the metric used to evaluate efferent innervation. Both Kobrina et al. (2020) and Grierson et al. (2022) observed a more pronounced age-related decline in their CBA/CaJ mice when MOC innervation was quantified as the number of terminals per surviving OHC (instead of immunolabeled area). Very interestingly, in both studies the numbers of efferent terminals per OHC fell below 1 at the most apical locations which strongly suggests that orphaned OHC, without any efferent innervation, occurred. This was pronounced in the apical regions of our old gerbils and was quantified here for the first time. Efferent innervation metrics were then normalized per innervated OHC in Figures 3B,D, 6, 7. If we instead normalized per surviving OHC (see Supplementary Figure S2), the overall difference between young-adult and old ears was larger and more clearly significant (18% loss in innervation area per OHC, n-way ANOVA: *F*(1,78) = 8.08, *p* = 0.0057; and 16% loss in terminal number per OHC, n-way ANOVA: *F*(1,73) = 16.04, *p* < 0.0005). Orphaned OHC have been anecdotally reported in young-adult rhesus monkeys and humans (Liberman and Liberman, 2019) as well as in old mice of several strains (Fu et al., 2010; Jeng et al., 2020; Grierson et al., 2022). It is currently unclear whether this phenomenon is particularly pronounced in gerbils or whether it has simply received little attention in other species.

Age-related OHC loss and an increase in orphaned OHCs also need to be considered when comparing age-related losses in the numbers of brainstem MOC neurons to peripheral metrics of OHC efferent innervation. For both old gerbils (Radtke-Schuller et al., 2015) and old CBA/CaJ mice (Vicencio-Jimenez et al., 2021), MOC neuron numbers were reduced by, on average, 31 and 36%, respectively, compared to young adults. These numbers are in the same ballpark as OHC losses (mice: Kobrina et al., 2020; Grierson et al., 2022) or OHC losses and orphaned OHCs combined (gerbil: this study), and are thus consistent with the more moderate reductions observed for the efferent innervation of surviving OHC.

We conclude that the extent of age-related changes in the efferent innervation of surviving OHCs varies between studies, and that some of this variation may be due to the as yet underappreciated phenomenon of orphaned OHCs (i.e., OHCs without efferent innervation). In the gerbil, the proportion of orphaned OHCs clearly increased with age, and spread further basally along the cochlea. Surviving OHCs that were still efferently innervated showed a tendency for only a small reduction in efferent innervation. This is consistent with previous suggestions that the loss of MOC innervation might precede OHC death (Jacobson et al., 2003; Zhu et al., 2007; Fu et al., 2010; Grierson et al., 2022).

### 4.5 Implications for efferent function in the aging cochlea

Counts of olivocochlear neurons in the brainstem clearly showed a substantial age-related decline of both LOC and MOC (Radtke-Schuller et al., 2015; Vicencio-Jimenez et al., 2021). Most interestingly, the olivocochlear efferents were differentially more vulnerable to neurodegeneration than either vestibular efferents and trigeminal motor neurons (which share a common descent with olivocochlear efferents), or brainstem neurones of the ascending auditory pathway (Radtke-Schuller et al., 2015; Vicencio-Jimenez et al., 2021). This implicates a specific trigger for age-related, auditory efferent loss. Data on peripheral innervation, such as those reported here, suggest that this loss occurs, to a large extent, in parallel with the loss of efferent target structures in the cochlea, primarily OHCs and type-I afferents. A likely aging scenario is thus that OHCs and type-I afferents die steadily with advancing age, taking their efferents with them. The surviving cochlear circuitry, however, remains, at least numerically, intact. This, in turn, predicts that it should be difficult to attribute age-related cochlear functional deficits specifically to a decline in efferent function.

For LOC, current evidence does not clearly support any specific sequence of age-related degeneration. Data from mice and humans that showed a more extensive loss of afferent, compared to efferent, synapses at comparable ages (see section 4.3) might suggest that the type-I afferents disconnect from the IHC first. For the gerbil, where we found no such discrepancy, this would then imply that LOC terminals degenerate quickly (perhaps more quickly than in mice and humans) together with their respective afferent dendrite, leaving the LOC circuitry that remains on the surviving afferents intact. On the other hand, there is evidence for subtle re-modeling of the LOC circuitry in aging cochleae that may not be obvious in common metrics of efferent innervation density. New axosomatic efferent contacts with IHC may be formed (Lauer et al., 2012) by LOC branches (Jeng et al., 2021), leading to the intriguing hypothesis that age-related loss of afferent synapses and dendrites may induce some of the thus orphaned LOC efferent branches to move up onto the IHC. Axosomatic efferent synapses on aging IHC have been shown to be functional, and inhibitory (Zachary and Fuchs, 2015). It currently remains unclear whether this is a general phenomenon across species, or even across mouse strains (Jeng et al., 2021), and whether it could be functionally adaptive. Understanding of LOC function and any potential age-related deficits is still in its infancy, and awaits the development of specific assays (reviewed in Lauer et al., 2022).

Indirect assays of MOC function, via their known suppression of otoacoustic emissions, showed age-related declines in both mice and humans, consistent with neurodegeneration (Kim et al., 2002; Jacobson et al., 2003; Zhu et al., 2007). Importantly, the decline began before there was any evidence of OHC degeneration, suggesting that the change was specific to the MOC efferent system. This hypothesis is supported by observations of mild losses of MOC innervation on surviving OHCs, and possibly an age-related increase in orphaned OHCs (see section 4.4) that, together, imply a sequence of degeneration, with efferents retracting first and OHCs dying shortly after. However, whether this truly causes a specific efferent dysfunction remains to be shown. Several technical issues with the rather indirect DPOAE suppression assay, as well as reduced afferent input to the neural feedback loops involved, due to age-related degeneration of type-I afferent neurons, remain valid concerns (reviewed in Guinan, 2018; Fuchs and Lauer, 2019). More direct measures of OHC functional aging revealed subtle changes in OHC size and membrane properties that, however, caused no functional deficits (Jeng et al., 2020). In summary, although there is (mainly anatomical) evidence that MOC degeneration may be the primary age-related event, closely followed by OHC death, any specific functional deficit associated with the initial MOC loss remains to be demonstrated. This hypothesized deficit should be larger, the longer is the delay between MOC loss and OHC death, leaving a more or less significant, transient population of orphaned OHCs that are no longer under efferent control.

## Data availability statement

The raw data supporting the conclusions of this article will be made available by the authors, without undue reservation.

## Ethics statement

The animal study was approved by Niedersächsisches Landesamt für Verbraucherschutz und Lebensmittelsicherheit (LAVES), Oldenburg, Germany. The study was conducted in accordance with the local legislation and institutional requirements.

## Author contributions

FS: Data curation, Formal analysis, Investigation, Methodology, Supervision, Validation, Visualization, Writing – original draft, Writing – review & editing. AP: Formal analysis, Investigation, Methodology, Validation, Writing – original draft, Writing – review & editing. CK: Conceptualization, Data curation, Funding acquisition, Methodology, Project administration, Resources, Supervision, Validation, Writing – original draft, Writing – review & editing.
