# Supplementary material for: Age-related changes in olivocochlear efferent innervation in gerbils
Source: Front Synaptic Neurosci. 2024 Jun 3;16:1422330. doi: 10.3389/fnsyn.2024.1422330 (PMC11180762; doi:10.3389/fnsyn.2024.1422330)
Supplement: Supplementary file 1 [file Data_Sheet_1.pdf]

Supplementary Material

Age-related changes in olivocochlear efferent innervation in gerbils

Friederike Steenken[1,2], Asli Pektaş[1], Christine Köppl[1,2,3*]

*** Correspondence:** Christine Köppl: Christine.Koeppl@uol.de

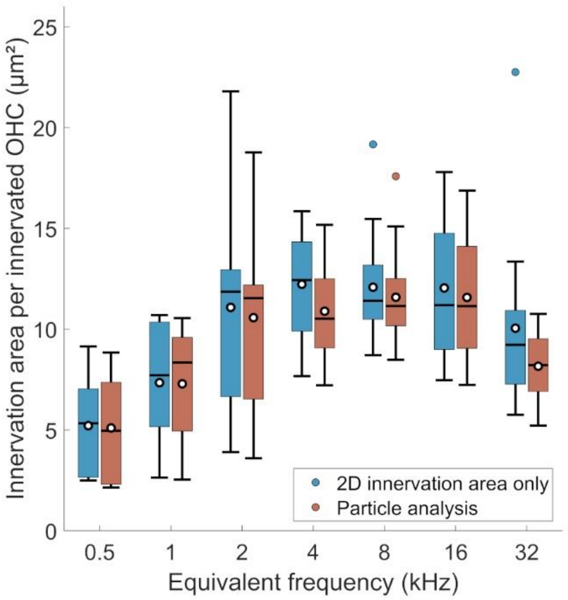

Supplementary Figure 1. Comparison of two metrics to quantify innervation area in the OHC region. The two methods and the rationales for them are detailed in section 2.5 of the paper. Briefly: 1) A z-projection using average pixel intensity was computed, followed by auto-thresholding using the algorithm "Li", to produce a binary image. The efferent innervation area was derived by multiplying the total ROI area with the fraction of synaptotagmin-labeled area within the ROI. 2) particle detection ("particle analyzer" plugin in ImageJ) was applied to the binarized image and the areas of all detected particles were summed. Particles were only included if they were larger than 2µm², holes were filled, and objects on edges were included. The ultimate results of these two slightly different procedures were not significantly different (mixed model ANOVA: $F(1,170) = 0.92$; $p = 0.34$) and are compared in the box plot. Blue boxes represent data obtained in all 20 gerbils (young-adult and old combined) with Method 1), red boxes show the result of Method 2). Box plots show the median (horizontal line), the 25th and 75th percentiles (upper and lower boundaries of the box), the data range without outliers (whiskers), and outliers (colored circles). Means are also depicted, as white circles within each box.

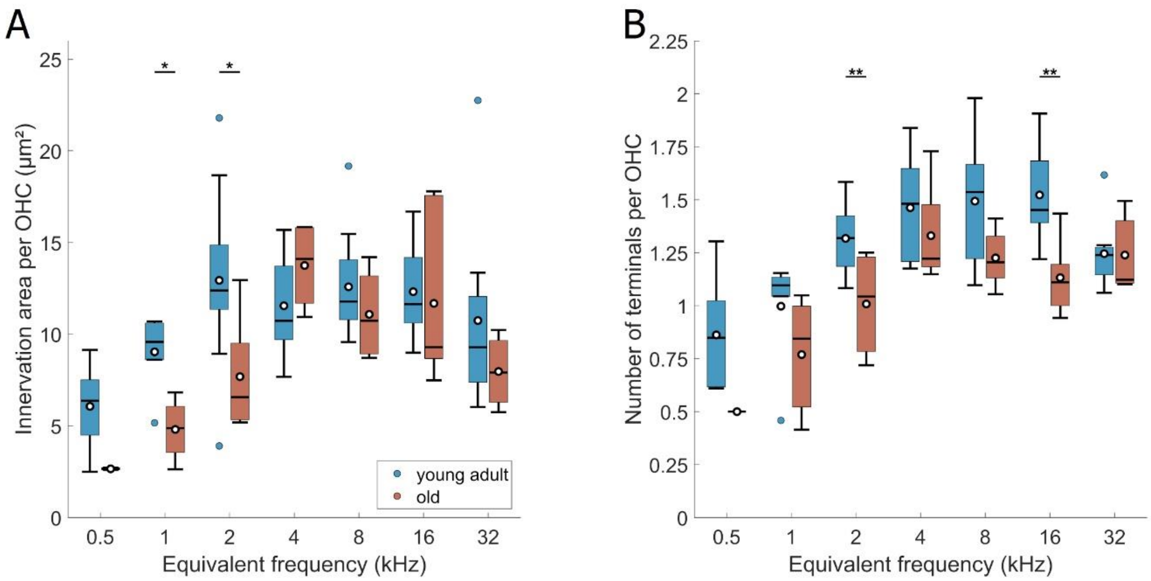

Supplementary Figure 2. Age-related changes of efferents in OHC region. (A) Innervation area and (B) number of terminals per OHC. Box plots show the median (horizontal line), the 25th and 75th percentiles (upper and lower boundaries of the box), the data range without outliers (whiskers), and outliers (colored circles. Means are also depicted, as white circles within each box. Boxes in blue and red display data from young-adult and old gerbils, respectively. Note that not every cochlear frequency included data for all 12 young-adult gerbils and 8 old gerbils (see Tables 1 and 2 in the paper). Asterisks denote significant differences between the age groups indicated by the respective line below (post-hoc tests; $*p < 0.05$, $**p < 0.01$).
